# *Mycobacterium doricum* Osteomyelitis and Soft Tissue Infection

**DOI:** 10.3201/eid1711.110460

**Published:** 2011-11

**Authors:** April C. Pettit, A. Alex Jahangir, Patty W. Wright

**Affiliations:** Vanderbilt University School of Medicine, Nashville, Tennessee, USA

**Keywords:** Mycobacterium doricum, tuberculosis and other mycobacteria, bacteria, osteomyelitis, soft tissue infection, orthopedic trauma

**To the Editor:** Infections with nontuberculous mycobacteria (NTM) are being increasingly identified. Several factors may contribute to this finding, including increased awareness of these organisms as pathogens, improved ability of laboratories to isolate and identify these organisms, and increasing prevalence (*1*). We describe a case of osteomyelitis and soft tissue infection with *Mycobacterium doricum* after trauma in a previously healthy adult.

A 21-year-old man sustained an open right femur fracture with gross contamination of the wound with dirt and gravel. The wound was irrigated and debrided, the fracture was fixed by intramedullary nailing, and the wound was closed.

Sixteen weeks later, pain, swelling, and erythema developed in the right thigh of the patient. Radiographs showed incomplete union of the fracture and possible loosening of the nailing hardware. Repeat irrigation and debridement of the right thigh was performed. Thirty milliliters of purulent material was drained. Bone was not visible, and the hardware was not removed or replaced. Gram staining of the purulent material showed 1 polymorphonuclear cell per oil-immersion microscopic field and no bacteria. Routine bacterial cultures were negative. Results of acid-fast staining were also negative. Three operative specimens were tested for acid-fast organisms. The patient was discharged and prescribed a 6-week course of vancomycin, 750 mg intravenously every 8 hours, and ciprofloxacin, 500 mg orally 2×/d.

Four weeks later, all 3 operative cultures grew an acid-fast bacillus, which was identified by DNA sequencing and high-performance liquid chromatography as *M. doricum* (Centers for Disease Control and Prevention, Atlanta, GA, USA). While in vitro susceptibility testing results were pending (*2*), recurrent swelling, erythema, and warmth developed in the patient at the previous injury site. A computed tomography scan showed multiple small abscesses adjacent to the nonunited fracture, an irregular periosteal reaction around the fracture site, and lucency surrounding the medullary rod, suggestive of osteomyelitis (Figure A1).

The patient was treated with irrigation and debridement of the abscesses and exchange of hardware. Two specimens were tested for acid-fast culture, 1 of which grew *M. doricum* after 3 weeks of incubation. The patient was discharged and empirically treated with amikacin, 1,250 mg/d intravenously for 3 weeks, and levofloxacin, 750 mg/d orally for 3 months, as therapy for infection with *M. doricum*, pending susceptibility testing results.

In vitro susceptibility results showed susceptibility to all drugs tested: MIC <1 μg/mL for amikacin, 0.25 μg/mL for ciprofloxacin, 4 μg/mL for clarithromycin, 2 μg/mL for ethambutol, <0.12 μg/mL for rifampin, <0.25 μg/mL for rifabutin, <0.5 μg/mL for streptomycin, and <0.12/2.4 μg/mL for trimethoprim/sulfamethoxazole (Associated Regional and University Pathologists, Salt Lake City, UT, USA). The antimicrobial drug regimen was changed to trimethoprim/sulfamethoxazole (800 mg of trimethoprim and 400 mg of sulfamethoxazole) orally 2×/d, and doxycycline, 100 mg orally 2×/d. After 10 months of therapy, the patient stopped taking these antimicrobial drugs. Six weeks after discontinuation of therapy, he had no signs or symptoms of recurrent infection.

*M. doricum* was first identified in a cerebrospinal fluid sample from a 50-year-old man with AIDS (CD4+ lymphocyte count 28 cells/mm^3^). *Cryptococcus neoformans* was also isolated from the same specimen. This patient was treated for *C. neoformans* infection with amphotericin B and 5-fluorocytosine but died 6 weeks later, before isolation of *M. doricum*. The isolate was susceptible to all antimicrobial drugs tested in vitro, including amikacin, azithromycin, clarithromycin, ciprofloxacin, clofazimine, ethambutol, isoniazid, ofloxacin, rifabutin, rifampin, sparfloxacin, and streptomycin (*3*).

The American Thoracic Society and Infectious Diseases Society of America have published guidelines on the diagnosis, treatment, and prevention of NTM diseases (*1*). However, there are no recommendations for treatment of *M. doricum* infection. At least 2 antimicrobial drugs are recommended for treating infections with NTM, and surgery is indicated for extensive disease, abscess formation, or contraindications/intolerance to medical therapy. When possible, it is recommended that foreign bodies be removed. For skin and soft tissue infections, >4 months of therapy is recommended, with extension to 6–12 months for cases of bone involvement (*1*). In this instance, trimethoprim/sulfamethoxazole and doxycycline were chosen because of availability of oral formulations, in vitro susceptibility testing results, and affordability given the patient’s lack of medical insurance.

Disease in the patient was eradicated by surgical debridement and prolonged antimicrobial drug therapy. We speculate that the organism was introduced from the soil at the time of the open fracture. Given increased awareness of NTM as pathogens and improvement in the ability of laboratories to isolate and identify these organisms from clinical specimens, this *Mycobacterium* species might be increasingly identified as a cause of disease. Additional reports of treating disease caused by *M. doricum* will be valuable so that in vitro susceptibility testing can be correlated with clinical responses, eventually enabling development of guidelines for therapy.

## Figures and Tables

**Figure A1:**
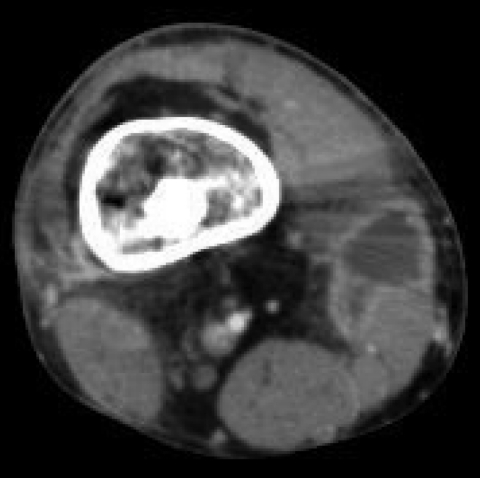
Computed tomography scan of the right lower extremity of a 21-year-old patient, showing abscess formation adjacent to nonunion of a right femur fracture.
